# Metastatic Hurthle Cell Carcinoma of the thyroid presenting as a Breast Lump: A Case Report

**DOI:** 10.1186/1477-7800-5-14

**Published:** 2008-05-27

**Authors:** Yahya Al-Abed, Emma Gray, Konrad Wolfe, Gavin W Watters, Jonathan M Philpott

**Affiliations:** 1 The Breast Unit, Department of Surgery, Southend University Hospital, Prittlewell Chase, Westcliff-on-Sea, Essex SS0 0RY, UK; 2 Department of Histopathology, Southend University Hospital, Prittlewell Chase, Westcliff-on-Sea, Essex SS0 0RY, UK; 3 Department of Ear, Nose and Throat, Southend University Hospital, Prittlewell Chase, Westcliff-on-Sea, Essex SS0 0RY, UK

## Abstract

**Background:**

Hurthle cell carcinoma of the thyroid is a rare form of thyroid cancer. It may present as a low grade tumour or can present as a more aggressive metastatic carcinoma. Hurthle cell carcinoma has the highest incidence of metastasis among all differentiated thyroid cancers. Most commonly haematogenous spread to lungs, bones and brain, however spread to regional lymph nodes is not uncommon. The breast is a rare site for metastasis from extramammary sources. We present the first case of breast metastasis from Hurthle cell carcinoma of the thyroid.

**Case presentation:**

We report a 77 year old lady who had total thyroidectomy and bilateral neck dissection followed by radiotherapy for a high grade metastatic Hurthle cell carcinoma of the thyroid. Ten months later she presented to the breast clinic with left breast lump and a lump at the left axilla. Fine needle aspiration cytology of the lumps and histology after wide local excision of the breast lump confirmed metastatic Hurthle cell carcinoma.

**Conclusion:**

The presence of breast lumps in patients with history of extramammary cancer should raise the possibility of metastasis.

## Background

The breast is a rare site of metastasis from extramammary sources. However, there are a number of documented cases with extramammary cancer sources of metastasis including gastric cancer, colon cancer, melanoma, cancer of the cervix, endometrial carcinoma and other rare cancers [1-3]. However, breast metastasis has never been documented following Hurthle cell carcinoma of the thyroid.

Hurthle cell carcinoma is a form of follicular cell carcinoma of the thyroid. It is rather unusual and a rare type of thyroid cancer. Although it forms less than 5% of all differentiated thyroid cancers, it accounts for the highest incidence of metastasis between all differentiated thyroid carcinomas [4]. It is reported that up to 34% of patients with Hurthle cell carcinoma develop metastatic disease. Liver, oesophageal, endobronchial, retroorbital, adrenal, abdominal, skull, cutaneous and other unusual sites of metastasis have been reported in literature.

We present the first case of breast metastasis from Hurthle cell carcinoma of the thyroid.

## Case Presentation

A 77 year old lady was referred to our breast clinic as she noticed a hard non painful lump at the upper inner quadrant of the left breast and another lump at the left posterior axillary fold for 3 weeks. Patient recalled having a recent trauma to the breast after having a fall. The patient had no previous breast problems and has no family history of breast cancer. Past medical history included having total thyroidectomy and bilateral neck dissection followed by radiotherapy 10 months earlier for high grade metastatic Hurthle cell carcinoma.

**Figure 1:**
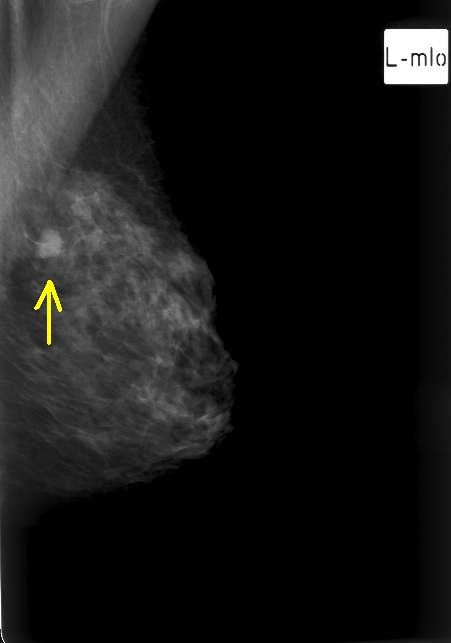
Left breast mammogram (L-mlo) view shows dense lobulated nodule at the upper inner quadrant (arrow).

On examination, there was bruising overlying the left breast due to the trauma the patient had earlier but no obvious lump, tethering of the skin or peau d' orange. Palpation of left breast revealed a mobile lump at the 11 o'clock position (upper inner quadrant). Examination of the left axilla confirmed a 2 cm lump overlying the lattismus dorsi. Mammography of the left breast showed an 11 mm dense lobulated nodule at the upper inner quadrant (figure 1). Ultrasound scan demonstrated the breast lesion as well as the axillary lesion as similar and relatively avascular and highly suspicious of metastasis from her thyroid tumour. Core biopsies and Fine needle aspiration cytology of both lesions confirmed secondary deposit of carcinoma.

**Figure 2:**
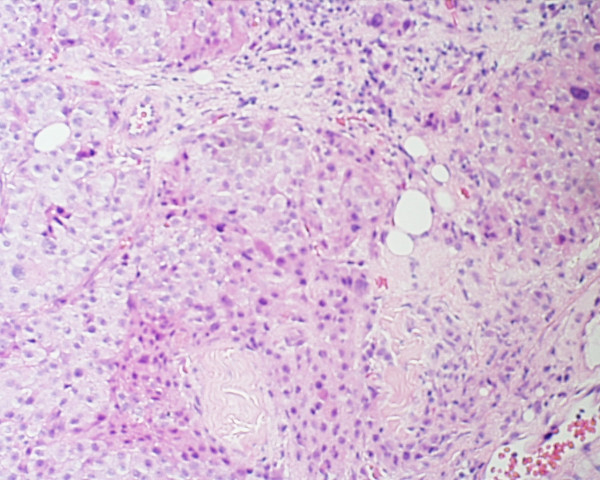
H & E section of the metastatic Hurthle cell carcinoma.

The patient underwent wide local excision of the left breast nodule and excision of the left posterior axillary fold lump. Histology of both lesions showed large pleomorphic cells with abundant eosinophilic cytoplasm which confirmed metastases from the previously diagnosed Hurthle cell carcinoma of the thyroid (Figure 2).

## Discussion

Metastasis to the breast from extramammary primary cancers is extremely a rare entity. The first reported case of metastasis to the breast was in 1903 by Trevithick who reported a reticulum cell sarcoma metastasis to the breast [5]. The incidence of metastatic cancers to the breast is approximately 1.2 – 2% [5,6]. There is a number of reported case series in the literature of metastatic disease to the breast from a primary cancer outside the breasts. Melanomas and neuroendocrine-like tumours being the most common types of metastasis to the breast from extrammamry primaries in adults [7]. Although it is rare, multiple diffuse and bilateral breast metastases have been reported following metastatic rhabdomyosarcoma to the breasts [8].

To our knowledge this is the first and only report of a case with metastatic Hurthle cell carcinoma of the thyroid presents as a breast lump. Among all differentiated thyroid cancers, patients with Hurthle cell carcinoma present with the highest incidence rate of metastasis [9].

Differentiating primary mammary disease from metastatic disease from extrammamry sources may be difficult on the clinical examination alone. Metastatic disease to the breast tends to be superficial and usually located at the upper outer quadrant [10]. The size of the deposit tends to correspond closely to the size detected on clinical examination while in primary breast cancer there is usually discrepancy between the size on clinical and mammographic examinations [11]. The reason for this is explained by the fact that primary breast cancer is associated with fibrous tissue proliferation and appears larger than the actual tumour itself.

Mammographic examinations in metastatic disease to the breast may vary. However, the majority of metastatic lesions to the breast appear to be well circumscribed round lesions and often lack spiculation and can some times be difficult to distinguish from benign breast lesions.

The presence of microcalcifications is often observed in primary carcinomas of the breast on mammography. Hajdu *et al *and Bohman *et al *suggested that presence of microcalcifications could potentially rule out metastatic disease to the breast [5,11].

## Conclusion

In conclusion, the presence of breast lumps in patients with history of extramammary cancer is usually helpful and should raise the possibility of metastasis. The diagnosis of metastatic disease to the breast is not always straightforward and as in primary breast cancer the triple assessment is necessary to establish the diagnosis.

## Competing interests

The authors declare that they have no competing interests.

## Authors' contributions

YA Literature searches, writing the paper.

EG Performed the surgical procedure, supervised and final revision of the article.

KW Examined the surgical specimen, wrote the pathological report and provided the histological photographic slides.

GW Performed original thyroid operation and involved in the after care of the patient.

JP Performed original thyroid operation and involved in the after care of the patient.
